# THE EFFECT OF MONOSIALOGANGLYOSIDE (GM-1) ADMINISTRATION IN SPINAL CORD INJURY

**DOI:** 10.1590/1413-785220162403160032

**Published:** 2016

**Authors:** TARCÍSIO ELOY PESSOA BARROS, FERNANDO FLORES DE ARAUJO, LUCAS DA PAZ HIGINO, RAPHAEL MARTUS MARCON, ALEXANDRE FOGAÇA CRISTANTE

**Affiliations:** 1. Universidade de São Paulo, Faculdade de Medicina, Department of Orthopedics and Traumatology, São Paulo, SP, Brazil; 2. Universidade de São Paulo, Faculdade de Medicina, Hospital das Clínicas, Instituto de Ortopedia e Traumatologia, São Paulo, SP, Brazil

**Keywords:** Spinal cord injuries, G(M1) Ganglioside, Outcome assessment (Health care).

## Abstract

**Objective:**

To evaluate the effect of monosialoganglioside (GM-1) in spinal cord trauma patients seen in our service who have not been treated with methylprednisolone.

**Methods:**

Thirty patients with acute spinal cord trauma were randomly divided into two groups. In Group 1, patients received 200 mg GM-1 in the initial assessment and thereafter received 100 mg intravenous per day for 30 days and Group 2 (control) received saline. Patients were evaluated periodically (at 6 weeks, 6 months, one year and two years), using a standardized neurological assessment of the American Spinal Injury Association / International Spinal Cord Society.

**Results:**

The comparative statistical analysis of motor indices, sensitive indices for pain and touch according to the standardization of ASIA / ISCOS showed that the assessments at 6 weeks, 6 months and 2 years, GM-Group 1 patients had higher rates than the control group regarding sensitivity to pain and touch, with no statistically significant difference from the motor index.

**Conclusion:**

The functional assessment showed improvement in the sensitive indices of patients treated with GM1 after post-traumatic spinal cord injury compared to patients who received placebo. ***Level of Evidence IV, Prospective Case Studies Series.***

## INTRODUCTION

The prevalence of spinal cord injury in Brazil due to external causes (accidents or violence) is high, about 71 new cases/year/million, accounting for 11.000 new cases/year, higher than the international data, less than 50/million. The cost generated by the morbidity and mortality of spinal cord injury, taking into account only primary care hospital expenses amounts to approximately US$ 95.000.^1^ ^,^ ^2^

After a spinal cord injury, a complex process of metabolic reactions ultimately leads to cell death and consequently, functional loss. The cellular necrosis at the site of injury due to mechanical stress is followed by secondary injury of apoptotic nature, that also affects the adjacent tissue through a sequence of neurochemical changes - the "reactive cascade".^3^ ^,^ ^4^

The primary injury, a mechanical one, is irreversible and the surgical decompression and mechanical stabilization can be applied in cases of unstable fractures with medullar injury.^5^ Currently, the therapeutic principle of medical treatment of spinal cord injury is directed to the reduction/inactivation of the secondary injury and to the attempt to promote axonal regeneration. Unfortunately, however, the current research efforts did not yet led to a pharmacological strategy with proven benefits, and only two drugs are used clinically: monosialoganglioside (GM-1) and methylprednisolone.^6^ ^-^ ^8^

One alternative that has been tested is the early administration of high doses of methylprednisolone. However, the evidences of its effectiveness are weak^6^ ^-^ ^10^ and the harmful effects of this substance for neuronal regeneration are already know, such as inhibition of immune cell activity,^11^ neutropenia, exacerbation of post-ischemic necrosis and inhibition of axonal sprouting,^12^ as well as respiratory complications, sepsis and gastrointestinal bleeding.^6^

Gangliosides are sialic acid derivatives of endogenous glycolipids present predominantly in the cell membrane in the central nervous system (CNS). GM-1 is already a therapeutic option for treatment of CNS injuries with antineurotoxic, anti-inflammatory and neuroprotective effects, being essential in neuronal excitability.^13^ Moreover, it promotes the development, growth, differentiation and neuronal maturation and reduces the intensity of the Waleriana degeneration.^13^ ^,^ ^14^ Research involving GM-1 in humans have shown improvement of locomotor function in spinal cord injury victims,^15^ but the interpretation of these results is complicated by the fact that methylprednisolone has been used prior to GM-1 administration.^14^ ^,^ ^15^

The purpose of this study was to evaluate the effect of monosialoganglioside (GM-1) in spinal cord trauma patients seen in our service that have not been previously treated with methylprednisolone.

## MATERIALS AND METHODS

**Figure 1:**
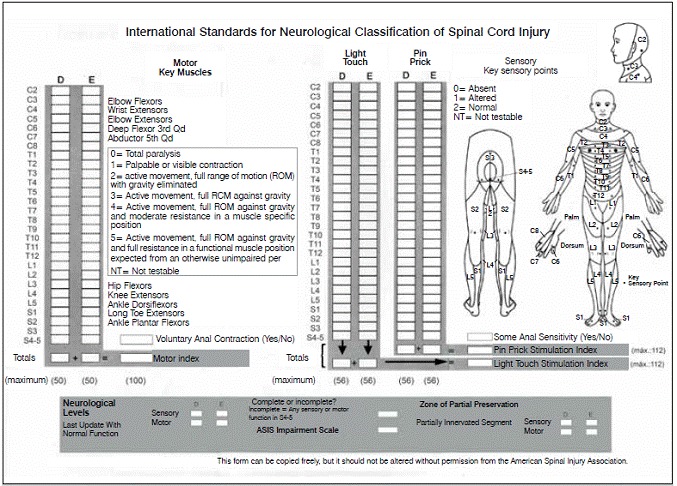
Standardization of the neurological evaluation of injuries

This is a controlled, double blind study aimed to compare the use of monosialoganglioside (GM-1) *versus* placebo in patients suffering from spinal cord injury. It was held at the Spinal Cord Trauma Unit of *Instituto de Ortopedia e Traumatologia do Hospital das Clinicas da Faculdade de Medicina da Universidade de São Paulo* between January 2000 and February 2001. Every institutional and ethical norms were followed. We included 30 consecutive patients aged 18-50 years old, admitted between 8 and 72h after trauma, with closed injuries of the spine from C4 to T10 with associated neurological deficit classified as: A, complete injury; B, sensitive preservation only; C, non-functional motor preservation; D, functional motor preservation; and E, without any deficit, according to the American Spinal Injury Association/International Spinal Cord Society (ASIA/ISCOS) standardization (Figure 1). Patients under 18 and over 50 years old, admitted within less than 8h and over 72h after trauma, with spinal trauma but without any neurological deficit, with open/exposed injuries, with spinal cord injuries above C4 or below T10, with altered level of consciousness making impossible to assess neurological deficit in a timely manner to be included in the protocol, with neurological deficit from other causes prior to spinal cord injury were excluded from the sample.

Patients were randomized into two groups by drawing lots in sealed envelops. GM-1 group received 200 mg medication intravenously in primary care and thereafter 100 mg IV per day for 30 days. The control group received saline as placebo.

Patients were evaluated periodically (at 6 weeks, 6 months, one year and two years), through an ASIA/ISCOS standardized neurological assessment, consisting of sensory and motor evaluation. The first one is divided into pain (nip) and light touch (cotton). Both are ranked as (2) Normal, (1) impaired, and (0) with no sensitivity. For each type of sensitivity (superficial touch and pain) a total score of 56 points is produced (0 to 2 for each of the 23 defined dermatomes). The overall sensory index is 112 for each tested sensitivity. Motor evaluation is ranked 0-5, according to the Medical Research Council Scale for Muscle Strength: (0) paralysis; (1) movements present without overcoming gravity; (2) movement in full amplitude overcoming gravity; (3) full range movements overcoming gravity, (4) against some resistance; and (5) against total resistance. If a muscle could not be tested, it was defined as NT (not tested). The sensory and motor indexes are the numerical sum of the scores, reflecting the degree of neurological disability associated with spinal cord injury.

Statistical analysis was based in mean, median and standard deviation of the data. The results were compared using mixed effect models with two factors: group and assessment week, considering the repetition of measurements over the weeks. The effect of the interaction between these factors was also evaluated. The association between the index obtained and the groups was assessed using Fisher's exact test. *p*-Values ​​less than 0.05 were considered as statistically significant.

## RESULTS

The groups were similar in terms of injury location (in the GM-1 group there were 10 cervical injuries and five thoracic injuries; in the control group there were nine cervical injuries and six thoracic injuries). The mean age was 32.1 years old in GM-1 group and 30.8 in the control group. The mean arrival time at the hospital after trauma was 24h in GM-1 group and 26h in the control group. Regarding gender, there were 14 men and one woman in both groups. Initial ASIA/ISCOS motor index was 39 in GM-1 group and 37 in the control group; as for sensitive ASIA/ISCOS pain index, it was 51 in GM-1 group and 53 in the control group; and touch sensitive index was 60 in GM-1 group and 61 in the control group.

The comparative statistical analysis of motor indexes, sensitive index for pain and sensitive index for touch, according to ASIA/ISCOS standardization, showed that in assessments at 6 weeks, 6 months and 2 years, patients in the GM-1 group showed higher rates than the control group regarding sensitivity to pain and touch, with no statistically significant difference for motor index.

## DISCUSSION

Recently, research in spinal cord injury shifted its focus from attempts to stop or slow the cascade of events of the secondary injury to effectively finding drugs that promote neuronal repair and regeneration. It has been known for some time that neuronal regeneration capacity, although somewhat reduced in the central nervous system as to the peripheral nervous system, recovers slowly and incompletely.^7^ ^,^ ^16^ The mortality rate in the first year after the acute phase of spinal cord injury ranges from 8 to 15% .^17^ ^,^ ^18^

Those patients have neural and morphological changes in the gastrointestinal system, obesity and its comorbidities, such as hypertension, heart disease, diabetes mellitus, decubitus ulcers, vascular disorders, tendinous muscular contractures, and sexual dysfunction. Chronic pain affects between 11 and 94% of these cases, substantially increasing the incidence of mental illness and difficulty of maintenance therapies.^19^ ^,^ ^20^ Although there is no consensus, GM-1 shows with to be a promising therapeutic option, evidence of its benefit both isolated or in combination with other physical, chemical or biological means have been found in the literature. Souza et al.,^21^ although not statistically significant, demonstrated the benefit of GM-1 in rats with experimental spinal cord injury, reaching higher rank in the assessment by the BBB score.

Santos et al.^22^ and Souza et al.^23^ presented preliminary results favorable to low temperature laser associated to GM-1 in spinal cord and peripheral nervous system injuries, while others have failed to demonstrate statistical significance regarding neurological recovery or show any difference between the results by evaluating the evoked potential.

Hyperbaric oxygen therapy was also investigated as a GM-1 enhancer in rats with experimental spinal cord injury and, although not statistically significant, has showed benefits in neurological recovery with GM-1, and this benefit was anticipated by hyperbaric oxygen therapy.^24^ Marcon, ^25^ in his thesis, also showed that GM-1 and erythropoietin have therapeutic effects on motor and electrophysiological function and axonal regeneration in Wistar rats with experimental spinal cord injury. Moreover, this author found out that that the combination of both substances potentiates its effect.

GM-1 appears to be reliable in all cases of spinal cord injury. In our study we showed significant differences among the groups. From the sixth week after spinal cord injury, patients who received GM-1 had a significant neurological improvement (according to ASIA/ISCOS standardization) as compared to patients who received placebo. The evidence that the performance was improved up to two years, makes one think that in acute and subacute spinal cord injuries benefit from GM-1. It would be interesting to reevaluate those benefits with a larger sample and other bone marrow analyzes, such as electron microscopy and specific methods of nerve regeneration, besides new experimental studies employing associations of growth factors and neural protectors, among other molecules, in search for more significant results in spinal cord injured patients.

## CONCLUSION

Functional assessment of patients shows improvement in sensory indexes with GM1 after post-traumatic spinal cord injury as compared to patients treated with placebo.
